# Rethinking Repeat Surgery for Median Neuropathy at the Carpal Tunnel

**DOI:** 10.1016/j.jhsg.2023.06.006

**Published:** 2023-07-18

**Authors:** David Ring, Jack G. Graham, Kyle J. Plusch, Bryan A. Hozack, Asif M. Ilyas, Jonas L. Matzon

**Affiliations:** ∗ Dell Medical School, The University of Texas at Austin, Austin, TX; † Division of Hand & Wrist Surgery, Rothman Orthopaedic Institute at Thomas Jefferson University, Philadelphia, PA

To the Editor:

The study by Graham et al^1^ is worthy of attention. The study inferred that patients and surgeons should think twice before considering repeat release of transverse carpal ligament (TCL). It is notable that among 24 of 11,847 hands that underwent repeat release, only 1 was performed by a different surgeon (suggesting that people do not look elsewhere for repeat release) and only 1 was ascribed to incomplete release.^2^

In three patients, the surgical report was not available. Among the other 21 repeat releases, 33 surgical findings were used to support the repeat surgery. Twenty-two of those findings were arguably expected findings after surgery for severe idiopathic median neuropathy at the carpal tunnel, including nine instances of “reformed” or “scarred/thinned” TCL (the TCL heals and is indistinguishable from native ligament^3^), scarring (universal with surgery), and flattening or hourglass-like constriction of the nerve (a universal feature of advanced median neuropathy at the carpal tunnel). The remaining eight findings were subjective, imprecise, and unreliable, including “synovial hypertrophy” in five, “constrictive band of distal forearm fascia” in two, and “incomplete release of the TCL” in one. It is possible that these findings might amount to confirmation bias and avoidance of the moral distress and psychologic discordance of performing a surgery that might have been unnecessary.^4^

Difficult recovery after carpal tunnel release is common (approximately 1 in 4 in a recent study^5^); however, the surgeons in the current study almost never performed repeat surgery. Resisting the pressure to act can be a matter of moral courage and fortitude.

It is also notable that among 16 hands that underwent repeat electrodiagnostic testing, only 3 were worse than they were prior to surgery. It would be helpful to know when the test was performed relative to the surgery. It is possible that the neuropathy worsened in the interval between the test and surgery. This could account for the postoperative measures being worse rather than persistent pathophysiology that might benefit from intervention. It is notable that no repeat surgery was performed for worsening of pathophysiology.

Presumably, the repeat surgeries were performed because of concerns about symptoms. The only factor that was correlated with repeat release was comorbid psychiatric diagnosis. Feelings of distress (concern) are associated with more intense symptoms.^5^

The current study informs patients and hand surgeons that carpal tunnel release is a surgery that is almost never repeated or revised. It is possible that none of the 24 repeat releases provided benefit. A blinded, randomized, simulated-surgery controlled trial of repeat release could determine whether perceived benefits derived from repeat release are due to nonspecific effects such as meaning and context (placebo) effects and regression to the mean rather than specific treatment of pathophysiology.

Until then, it might be wise to approach repeat release with caution. For instance, one could perform two postoperative electrodiagnostic tests separated by at least 4 months demonstrating notable worsening of pathophysiology over time. In other words, reproducible, objective, experimental evidence of ongoing pressure on the nerve should be considered.
